# Optimising Mg-Ca/PLA Composite Filaments for Additive Manufacturing: An Analysis of Particle Content, Size, and Morphology

**DOI:** 10.3390/ma17122983

**Published:** 2024-06-18

**Authors:** Hyeonseok Kim, Tom McKenna, Eoin O’Cearbhaill, Mert Celikin

**Affiliations:** School of Mechanical and Materials Engineering, University College Dublin, D04 C1P1 Dublin, Ireland; tom.mckenna@ucd.ie (T.M.); eoin.ocearbhaill@ucd.ie (E.O.)

**Keywords:** additive manufacturing, material extrusion, filament, magnesium alloy, biomaterials, particle content, particle size, particle morphology, viscosity

## Abstract

Low-temperature additive manufacturing of magnesium (Mg) alloy implants is considered a promising technique for biomedical applications due to Mg’s inherent biocompatibility and 3D printing’s capability for patient-specific design. This study explores the influence of powder volume content, size, and morphology on the mechanical properties and viscosity of polylactic acid (PLA) matrix composite filaments containing in-house-produced magnesium–calcium (Mg-Ca) particles, with a focus on their application towards low-temperature additive manufacturing. We investigated the effects of varying the Mg-Ca particle content in a PLA matrix, revealing a direct correlation between volume content and bending strength. Particle size analysis demonstrated that smaller particles (D50: 57 μm) achieved a bending strength of 63.7 MPa, whereas larger particles (D50: 105 μm) exhibited 49.6 MPa at 20 vol.%. Morphologically, the filament containing spherical particles at 20 vol.% showed a bending strength that was 11.5 MPa higher than that of the filament with irregular particles. These findings highlight the critical role of particle content, size, and shape in determining the mechanical and rheological properties of Mg-Ca/PLA composite filaments for use in material extrusion additive manufacturing.

## 1. Introduction

Additive manufacturing has gained attention as a versatile technology, enabling the use of various materials, such as biodegradable metals, polymers, and ceramics, to produce patient-specific implants [1,2,3]. Among these materials, magnesium (Mg) alloys are emerging as promising biomedical materials due to their unique benefits in terms of biocompatibility, biodegradability, and mechanical properties [4,5]. Additionally, a distinctive feature of Mg alloys is their biodegradability in a physiological environment. This allows the implant to degrade gradually, potentially being replaced by the patient’s bone tissue, thereby reducing the need for secondary removal surgeries [6,7]. Moreover, Mg alloys exhibit elastic moduli (around 45 GPa) comparable to those of cortical bone (30 GPa). This similarity provides adequate support during healing, preventing stress shielding that could lead to bone loss [8]. Mg alloys hold significant potential for next-generation biomedical applications, especially in the fabrication of personalised implants using AM. However, the high reactivity of Mg alloys presents safety risks and significant challenges for high-temperature AM techniques such as laser powder bed fusion (LPBF). High temperatures involved in the LPBF process can cause rapid oxidation, leading to the formation of oxide particles, porosity, and variations in composition and microstructure. These changes degrade mechanical performance and biocompatibility [9,10,11].

**Figure 1:**
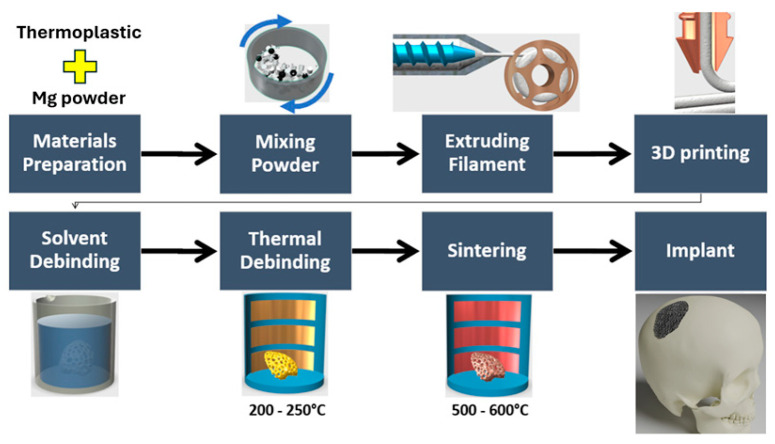
Schematic diagram of material extrusion additive manufacturing of metallic components.

Material extrusion-based AM (MEAM) as a low-temperature manufacturing method has been explored to address the challenges of high-temperature processes. Unlike processes that involve melting the metal powder, MEAM requires lower temperatures (<200 °C) during AM processing. Nevertheless, post-processing which involves solvent/thermal de-binding and a final sintering step is essential after producing the green parts via MEAM. The schematic diagram of the MEAM process is depicted in Figure 1. This process offers distinct advantages, such as cost-effectiveness [12] and user-friendly operation, making it a favourable option for various applications compared with other AM techniques. However, MEAM relies on thermoplastic filaments as the feedstock. This poses specific challenges, especially when incorporating powders into a filament [13]. A notable issue in MEAM is the brittleness of the filaments, which escalates with the powder content. Such brittleness compromises extrudability and printability during filament winding onto the spooler and feeding into the 3D printer [14]. To overcome this limitation, extensive research has been conducted to develop specialised filaments suitable for MEAM of Mg alloys. For instance, Antoniac et al. introduced pure Mg/PLA filaments incorporating additives for MEAM of Mg. Blending with vitamin E strengthened Mg particle adherence to the PLA matrix, improving filament integrity and curtailing Mg particle agglomeration [15]. Other studies indicated that integrating thermoplastic elastomer (TPE) and polypropylene-copolymer-polyethylene ameliorates filament flexibility and processability, resolving spooling and feeding issues [16,17].

Another important aspect to consider in the development of composite filament is the influence of particle size and morphology. Specifically, the utilisation of large 316L steel particles enhanced the strength of the filament constituting a main binder component of thermoplastic elastomer with a backbone of polyolefin, whereas smaller particles decreased the elongation and the viscosity of the filament [18]. Powder morphology, specifically the difference between spherical and irregular shapes, is another critical element in determining filament quality [19]. However, the influence of morphology can vary depending on the type of fillers employed, leading to distinct interactions with binders. For instance, when assessing the tensile strength, significant variations can arise based on the specific material under consideration. The resultant variations in mechanical properties are profoundly influenced by the chemical and physical interactions between the particles and the matrix [20]. Understanding the effects of metal filler morphology, size, and content has gained increasing attention due to their significant implications in the extrudability and printability of filaments containing metal powder.

Stainless steel (316L), titanium-based alloy (Ti6Al4V), copper (Cu), NdFeB, and zirconia have been investigated for the effects of morphology, size, and content of metal filler [18,19,20,21]. However, the effects of morphology, size, and content of magnesium (Mg) alloy powder on filament quality remain ambiguous. Antoniac et al. worked with an Mg/PLA composite as well, but the effects of powder content, size, and morphology were not considered [15]. Kukla et al. looked at the effects of size and morphology, but the materials were not adequately controlled and the morphology of the powder was not quantified [20]. The primary objective of this study is to establish a baseline for the development of high-quality 3D-printed Mg components via low-temperature AM technology. Specifically, this research seeks to determine the effects of particle size, morphology, and volume content of Mg-Ca powder on the extrudability of PLA-based composite filaments. To achieve this, we create different Mg-Ca powder morphologies via dry milling and ball milling, and separate the powders of two distinct sizes by sieving.

## 2. Materials and Methods

Mg-Ca/PLA composite filaments as feedstocks for material extrusion-based AM were produced by a filament maker, Composer 350 from 3devo (Utrecht, The Netherlands). The filaments consist of Mg-Ca-based alloy powder and a polylactic acid (PLA)-based blend composed of 90 wt.% PLA (Utrecht, The Netherlands), 9 wt.% paraffin wax (thermos scientific), and 1 wt.% stearic acid (Sigma-Aldrich, Burlington, VT, USA). The Mg-Ca powder was in-house produced from Mg–20Ca ingot in wt.% (Shanghai Xinglu Chemical Tech, Shanghai, China) via a dry milling process without cutting fluid. The dry milling process was executed according to the following parameters: 1 mm depth of cut and 200 mm/min travel speed, achieving 5 mm every 24 s using Emill milling equipment and NDrill [22]. Half of the dry-milled powder was further processed to reduce particle size and to spherodise the particle via ball milling, DECO-PBM-V-0.4L (Changsha Deco Equipment Co. Ltd., Yueyang, China), under the parameters shown in Table 1.

Both dry-milled and ball-milled powders were subsequently sieved into two size ranges: (i) 125 μm to 88 μm; and (ii) 88 μm to 25 μm. The segregated Mg-Ca powder, varying in size and morphology, was then mixed with PLA pellets at three different volume ratios: 5 vol.%; 10 vol.%; and 20 vol.% as summarised in Table 2.

The composite filament was produced by feeding the mixtures of Mg-Ca powder and PLA-based blend into the Composer 350 hopper. To make the mixture, paraffin wax (9 wt.%) and stearic acid (1 wt.%) were melted at 100 °C on a hot plate, which were then blended with sieved Mg-Ca powder. PLA pellets (90 wt.%) were subsequently added to the heated mixture at 190 °C and stirred manually. After mixing, the mixture was cooled to 20 °C. The filament maker utilised for this study was the Composer 350 from 3devo (Utrecht, The Netherlands). Operating parameters for the filament maker included a screw speed of 3.5 RPM and temperature settings of T1 = 170 °C, T2 = 190 °C, T3 = 185 °C, and T4 = 170 °C. The output nozzle diameter was 1.75 mm, and the ambient temperature during production was kept at 15 °C. Additionally, fan-assisted cooling was employed during extrusion to ensure filament solidification.

PLA pellets before extrusion were analysed via DSC using Netzsch DSC 214 polyma equipment (Selb, Germany). DSC was performed between 20–300 °C at a heating rate of 10 °C/min under nitrogen (N) atmosphere with a gas flow rate of 60 mL/min.

Three-point bending tests were conducted to assess the mechanical properties of the extruded filaments. The flexural strength of the filaments was measured using LS materials testing machines from Lloyd Instruments (AMETEK STC, Bognor Regis, UK) at 15 °C. The tests were conducted on filaments with a 1.75 mm diameter and a 15 mm point bending span until fracture occurred. The standard used for the three-point bending tests is ASTM D790 [23], which specifies the dimensions for specimens, including thickness, width, and length. However, our aim was to evaluate the flexural strength of a 1.75 mm filament, which is not covered by ASTM D790. Therefore, while we followed the standard for general guidelines, we adapted it to suit the shape of our filament.

The viscosity measurements of the Mg/PLA composites, integrated with the blend, were conducted using a modular compact rheometer (MCR 302e, Anton Paar, Graz, Austria) according to the ASTM D4440-15 [24]. The experiments were performed at a controlled temperature of 190 °C across a shear rate range from 0.01 to 10 s^−1^. A parallel plate measuring system was employed, with the plates having a diameter of 12 mm.

Scanning electron microscopy (SEM) was used to examine the microstructure and powder distribution in the extruded filaments with different powder contents. Analyses were carried out using an SEM TM4000 Plus (HITACHI, Tokyo, Japan) at an accelerating voltage of 10 kV. Several regions along the filament length were imaged at magnifications ranging from 500× to 5000×.

A digital microscope was used to examine the fracture mechanism of Mg-Ca/PLA composite filaments after the three-point bending test. Analyses were carried out using a VHX-5000 (Keyence, Osaka, Japan). The 3D topological images and 2D microscopic images were analysed using VH-Z500R (Keyence, Japan), which is a high-resolution zoom lens at 500× magnification.

**Figure 2:**
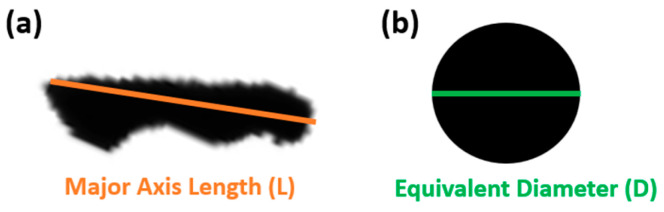
(**a**) The particle’s shape. (**b**) The circle which shares an equivalent area with the particle’s shape.

The size and morphology of Mg-Ca particles were determined using a laser diffraction (LD) analyser (SYNC of MICROTRAC, Duesseldorf, Germany). Each specimen’s assessment incorporated data from over 1000 particles. Beyond generating numerical data, the LD also produced 2D-images of individual particles. For enhanced accuracy in comparing powder morphology, additional analysis was conducted using a Python-based methodology on the 2D particle images sourced from the LD. Quantitative details regarding the particle roundness were obtained via a Python script, which streamlined the process of extracting roundness from 2D datasets. In Figure 2, the area of circle (Figure 2b) corresponds to the area of particle (Figure 2a). The orange line denotes the major axis length of particle (Figure 2a), whereas the green line on circle (Figure 2b) represents the equivalent diameter of a circle with the same area as particle (Figure 2a). The roundness was calculated as the ratio of the equivalent diameter to the major axis length (Equation (1)).

(1) $$Roundness=\frac{Equivalent\;Diameter\;(D)}{Major\;Axis\;Length\;(L)}=\frac{\sqrt{\frac{4A}{\pi }}}{L}$$

Consequently, merging LD data with Python-driven image analysis provided a more in-depth morphological exploration of the Mg-Ca particles.

## 3. Results and Discussion

### 3.1. Thermal Properties

**Figure 3:**
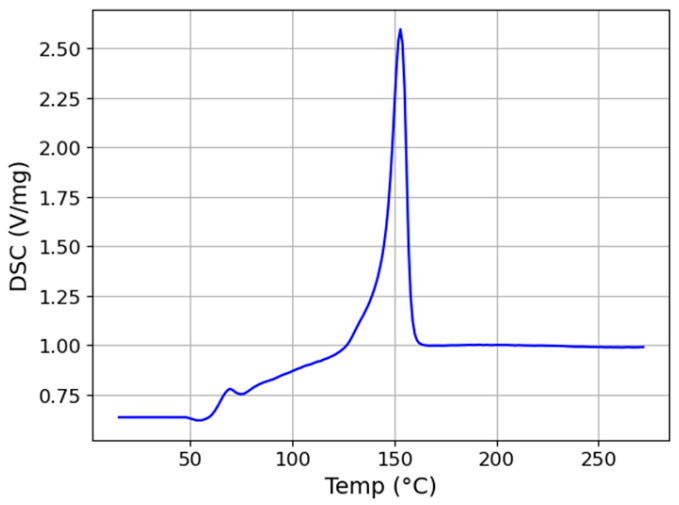
DSC results of PLA pellets.

**Figure 4:**
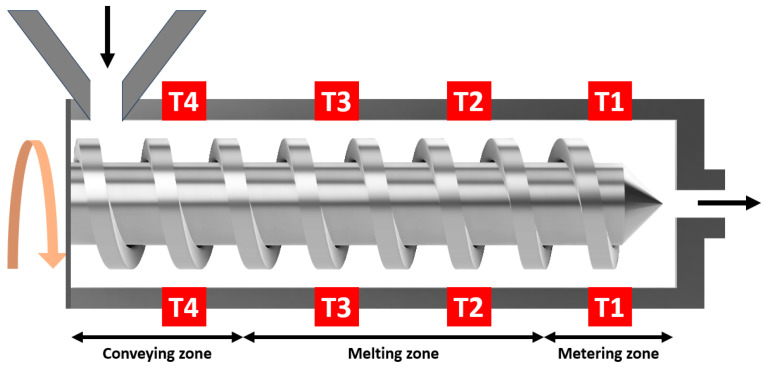
A schematic diagram of filament maker.

The DSC result, as shown in Figure 3, indicates that the Tm of raw PLA pellets is approximately 153 °C, consistent with the PLA supplied by NatureWorks LLC [25]. As depicted in Figure 4, the single screw filament maker consists of conveying, melting, and metering zones, each serving a specific function. The heating point in the conveying zone, near the hopper, should be kept below the Tm of PLA pellets. Excessive heating could liquefy the polymer, preventing it from being pushed into the melting zone. Within the melting zone, the temperature must surpass the Tm to ensure the PLA fully melts and effectively blends with the Mg-Ca powder. Elevating the temperature above 200 °C might initiate PLA molecular chain breakdown, potentially compromising the filament’s mechanical properties [26]. Moreover, the temperature of the metering zone should be optimised to consistently maintain the filament’s thickness. The second and third heating points in the melting zone, as shown in Figure 4, are the most critical, since the temperature of the melting zone significantly affects the filament’s mechanical properties and the blend’s quality.

### 3.2. Optimisation of Extrusion Process

**Figure 5:**
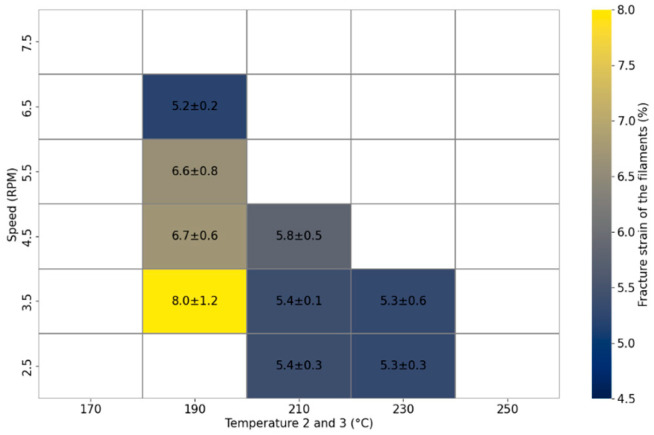
Elongation of PLA filaments at breaking point as a function of screw temperature and speed in filament maker.

The correlation between extrusion speed, temperature, and the resulting ductility of PLA filaments is depicted in Figure 5. Among the tested conditions, the filament fabricated at 3.5 revolutions per minute (RPM) and 190 °C exhibited the highest ductility, recorded as 8%. It is recognised that the mechanical properties of PLA, such as elongation, are influenced by factors including molecular weight and crystallinity [27]. Elevated temperatures, particularly those beyond 200 °C, initiate the degradation of PLA, leading to a reduction in molecular weight and, consequently, a decrease in elongation [26]. On the other hand, it can be stated that the crystalline structure of PLA is affected by the cooling rate. A slower rate of cooling permits more time for PLA to develop a crystalline structure, which is typically associated with diminished elongation [28]. However, at 190 °C, an increase in RPM results in decreased elongation due to the incomplete melting of the PLA pellets. When the temperature surpasses 210 °C with an RPM exceeding 5.5, or when it exceeds 230 °C with an RPM greater than 4.5, PLA exhibits liquid-like behaviour, rendering it unsuitable for filament formation. In contrast, at temperatures below 190 °C, PLA is not fully melted, which also hinders its ability to be formed into filaments. Consequently, the process of forming PLA into filaments is compromised at both ends of the temperature spectrum: excessive temperatures result in a liquefied state, whereas insufficient temperatures prevent the complete melting of the polymer. This emphasises the critical need for precise control of temperature and RPM during filament extrusion to ensure the effective production of PLA filaments.

### 3.3. Powder Size and the Size Distribution Analysis

**Figure 6:**
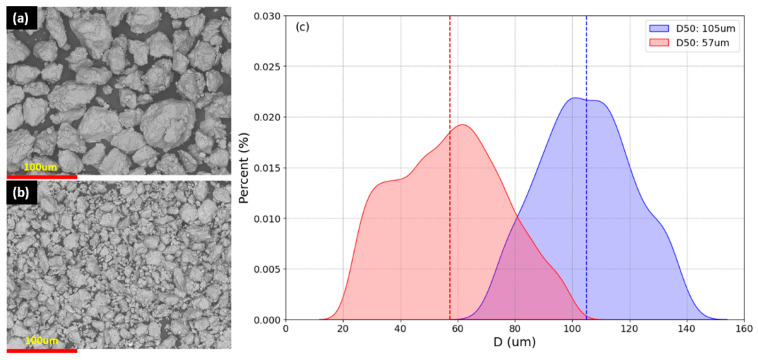
SEM image of ball-milled and sieved (**a**) D50: 105 μm particles, (**b**) D50: 57 μm particles, and (**c**) size distribution of D50: 105 μm and D50: 57 μm particles.

The scanning electron microscopy (SEM) images depicted in Figure 6a,b demonstrate a notable difference in the size ranges of two Mg-Ca powder samples: ball-milled and sieved. The particle size distribution for each sample, as determined by laser diffraction (LD) measurements, is quantified in Figure 6c using a histogram representation. The median particle size (D50) for the coarser powder sample is indicated by the blue dashed line and is measured as 105 μm in Figure 6c, while the D50 for the finer sample is marked by the red dashed line and is measured as 57 μm. For the sample with D50 of 105 μm, the 90th and 10th percentile sizes (D90 and D10) are calculated to be 127.9 μm and 83.6 μm, respectively. In contrast, the sample with D50 of 57 μm exhibits D90 and D10 at 83.2 μm and 31.4 μm, respectively, indicating a distribution overlap. The standard deviations are computed to be around 15.9 μm for the D50: 105 μm sample and 18.8 μm for the D50: 57 μm sample.

### 3.4. Powder Morphology Analysis

**Figure 7:**
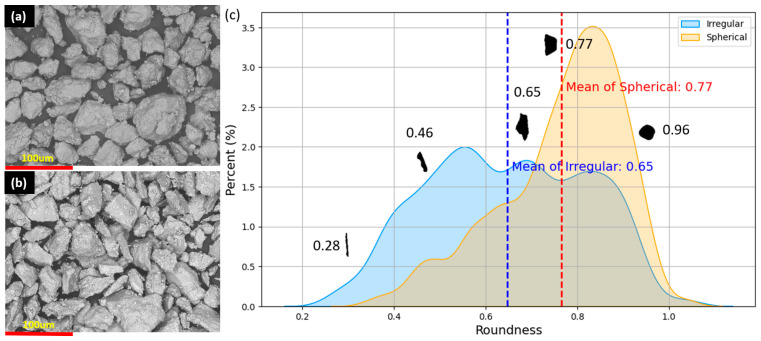
SEM image of (**a**) spherical particles, (**b**) irregular particles, and (**c**) roundness of spherical and irregular shape particles from laser diffraction analysis and Python image analysis.

SEM images reveal that dry-milled and sieved Mg-Ca powder particles exhibit a distinctly rough and irregular surface topology, in contrast to the smoother surface of ball-milled and sieved Mg-Ca powder particles (Figure 7a,b). Despite similar size characteristics, the particles display notable differences in roundness, as demonstrated in Figure 7c. This figure, derived from laser diffraction (LD) and Python-assisted analysis, illustrates the disparity in sphericity between spherical and irregular shapes, with a roundness value nearer to 1 denoting higher circularity. The mean roundness for spherical particles is calculated at 0.77, compared to 0.65 for irregular particles. Standard deviations are determined to be 0.13 for spherical and 0.17 for irregular particles. However, maximum roundness values occasionally exceed the theoretical maximum of 1. Such inaccuracies can be attributed to the limitations inherent in pixel-based imaging.

### 3.5. Microstructure Characterisation of Composite Filament Cross-Sections

**Figure 8:**
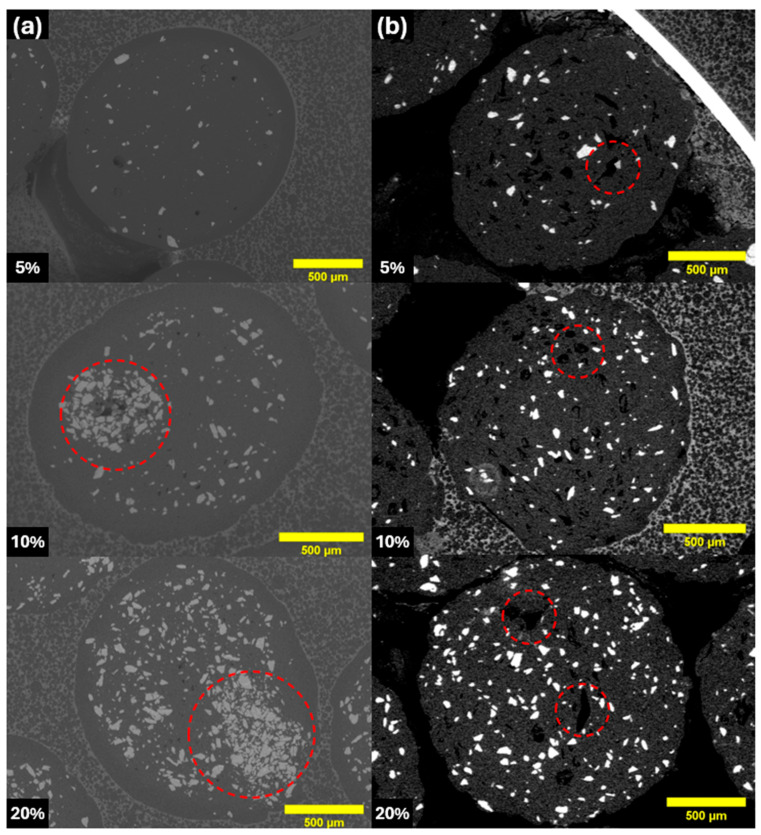
(**a**) Microstructure of spherical Mg-Ca powder (D50: 105 μm) composite filaments using PLA at Mg-Ca powder content of 5 vol.%, 10 vol.%, and 20 vol.%. (**b**) Microstructure of spherical Mg-Ca (D50: 105 μm) composite filaments using PLA blended with paraffin wax and stearic acid at Mg-Ca powder content of 5 vol.%, 10 vol.%, and 20 vol.%.

Figure 8a displays the microstructure of cross-sections of the filaments produced using only Mg-Ca powder and PLA, which were successfully manufactured without any porosity in the filament. However, localised agglomerations of Mg-Ca particles, highlighted by red circles in Figure 8a, were observed within the filament matrix at Mg-Ca powder content of 10 vol.% and 20 vol.%. These agglomerations are problematic as they not only reduce the mechanical strength of the filament but also cause clogging issues in the 3D printer’s nozzle by impeding material flow during extrusion. To address the issue of agglomeration [29,30], a novel blend, comprising 90 wt.% of paraffin wax and 10 wt.% of stearic acid, was integrated into a PLA matrix. The incorporation ratio in the composite was maintained at a 90 wt.% PLA and 10 wt.% blend. As depicted in Figure 8b, the resultant filament incorporating this new blend demonstrates that the Mg-Ca particles are more dispersed, resolving the previous agglomeration problem. Nonetheless, upon addition of this blend into Mg-Ca/PLA composite filaments, porosity levels are observed to be higher within the filament. The pores are highlighted by red circles in Figure 8b. This issue was primarily attributed to the difference in the melting points of paraffin wax, stearic acid, and PLA; the former two have melting points of around 60 °C, which are significantly lower than PLA’s melting temperature of 153 °C. During the filament solidification process, the regions containing paraffin wax and stearic acid may remain in a liquid state, due to their comparatively lower melting points. As the filament cooled and solidified, these liquefied sections led to the creation of pores, resulting in a porous filament structure. Such porosity could potentially affect the filament’s mechanical properties and its performance during 3D printing.

### 3.6. Effect of Mg-Ca Volume Content, Particle Size, and Morphology on Flexural Strength of Mg-Ca/PLA Composite Filaments

**Figure 9:**
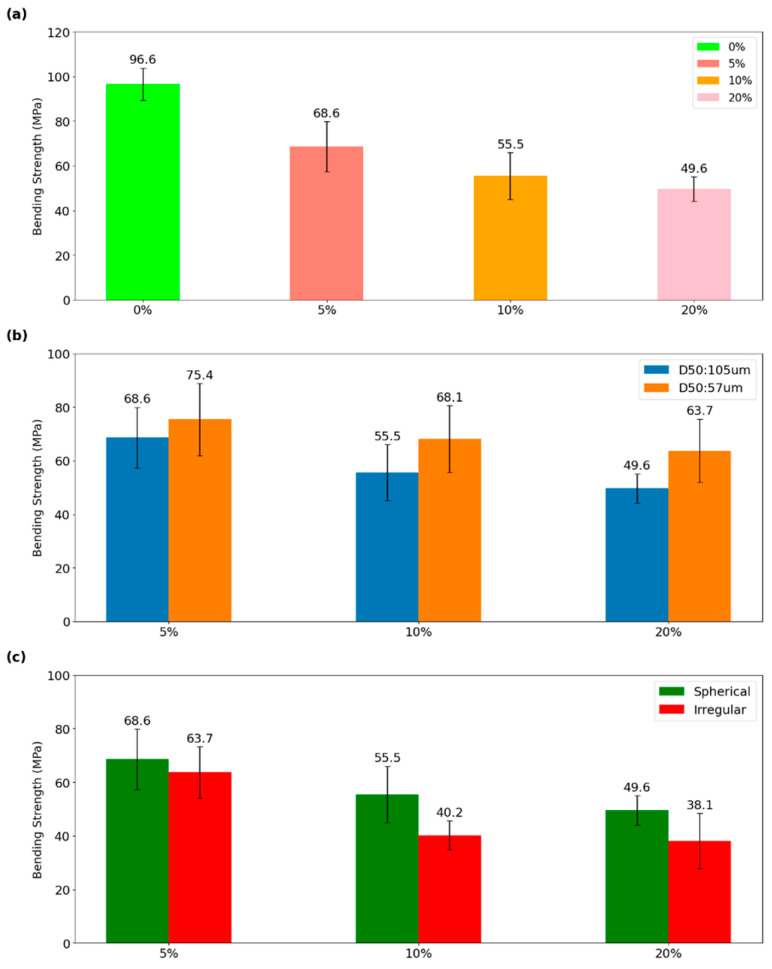
Bending strength of composite filaments of varying Mg-Ca powder in PLA blended with paraffin wax and stearic acid (**a**) as a function of the powder volume content, (**b**) as a function of the powder size, and (**c**) as a function of the powder morphology.

The three-point bending test results (Figure 9) conducted on Mg-Ca/PLA composite filaments revealed a pronounced decline in the bending strength of Mg-Ca/PLA composite filaments with increasing volume content of Mg-Ca powder. The PLA composite filament without Mg-Ca powder exhibited superior bending strength, diminishing progressively from 96.6 ± 7.2 MPa at 0% volume content to 49.6 ± 5.5 MPa at 20% volume content. This trend elucidates the direct correlation between the volume content of Mg-Ca and the flexural strength of the resulting filament. The reduction in flexural strength as the Mg-Ca content volume fraction increases is attributed to poor bonding between particles and matrix [31]. Poor adhesive qualities are typically observed between paraffin wax and PLA. Paraffin wax is a non-polar, hydrocarbon-based material with low surface energy, whereas PLA is, relatively, a polar thermoplastic with higher surface energy.

The differences in surface energies and polarities result in weak adhesion [32]. Furthermore, large standard error was observed, likely resulting from porosity induced by the differential solidification temperatures of paraffin wax and PLA during the extrusion process. To address this issue, one potential approach is to use non-polar polymers such as polypropylene (PP) or polyethylene (PE). These non-polar polymers can mix well with stearic acid and paraffin wax, enhancing both the miscibility and adhesion of the mixture. Alternatively, an advanced mechanical mixing method, such as a twin-screw extruder, can be utilized to effectively combine polar materials like PLA with Mg powder. This approach allows the continued use of polar polymers as the main backbone while achieving improved mixing and adhesion, thereby mitigating issues related to porosity and weak adhesion.

As the powder content increases, the composite filament filled with metal powder becomes stiff. This stiffness not only makes it difficult to wind the filament onto a spool during the filament making process but also leads to fracture problems when feeding the filament into a 3D printer. The filament must be straightened without breaking during the feeding process. To ensure smooth feeding into a 3D printer, filaments with higher bending strength are preferred.

The polymer matrix of printed parts should be removed through debinding process for practical application, followed by sintering to achieve a dense metal part. During sintering, reduction of porosity leads to shrinkage and densification, similar to the Metal Injection Moulding (MIM) process. In MIM, the typical powder content is approximately 64%. When the powder volume content exceeds 64%, the mould filling capability diminishes [33]. Additionally, the typical range of dimensional shrinkage is between 12 and 20% [13,34,35]. 20% of the maximum dimensional shrinkage indicate that the volumetric shrinkage is 48.8%. Therefore, a minimum powder loading of 51.2 vol.% is required for practical applications, assuming no porosity after sintering. However, in this study the powder content was only increased from 5% to 20% which is considerably lower than the practical use. The reason for this approach is to determine the effects and trends of Mg alloy powder content on the critical properties such as viscosity to develop a fundamental understanding and use this as a baseline for future work.

Figure 9b presents the effects of particle size on the bending strength of composite filaments. The data indicate that filaments with D50: 57 μm maintain higher bending strength values across all volume percentages compared to the filaments with D50: 105 μm. Specifically, at 5% volume content, the D50: 57 μm filaments exhibit a bending strength of 75.4 ± 13.5 MPa, whereas the D50: 105 μm filaments show a slightly lower strength level of 68.6 ± 11.3 MPa. This trend persists as the volume content increases, with a more pronounced decrease in bending strength for the larger particle size. The D50: 57 μm filaments resulted in greater resistance to bending strength, potentially due to effective stress distribution within the polymer matrix [31]. The increased surface area of these smaller particles facilitates a higher degree of interaction within the composite, leading to improved structural integrity [36].

Figure 9c shows the influence of particle shape on the bending strength of Mg-Ca/PLA composite filaments. It is evident that filaments containing spherical particles maintain a higher bending strength at a given volume content compared to those with irregular particles. For instance, at 5% volume content, filaments with spherical particles exhibit a bending strength of 68.6 ± 11.3 MPa, whereas filaments with irregular particles display a reduced strength of 63.7 ± 9.5 MPa. This difference becomes more pronounced with increasing volume content. At the 20% volume content, spherical particle filaments withstand a bending strength of 49.6 ± 5.5 MPa, in contrast to 38.1 ± 10.3 MPa for irregular particle filaments. The observed trend suggests that spherical particles may facilitate better stress distribution within the composite matrix due to their uniform geometry, leading to improved bending strength performance. Conversely, irregular particles could create stress concentrations due to their varied shapes, thus weakening the structural integrity [37].

### 3.7. Topology Analysis of Fracture Surface after the Three-Point Bending Test

**Figure 10:**
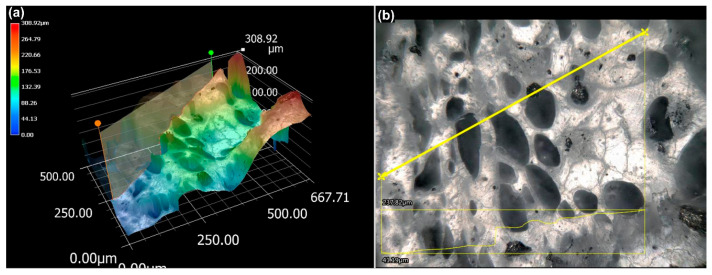
Fracture surface of 20 vol.% spherical Mg-Ca/PLA (D50:105 μm) of composite filament after three-point bending test: (**a**) 3D surface topology and (**b**) top-view image of (**a**).

Fracture surfaces of a 20 vol.% Mg-Ca composite filament upon the three-point bending tests were also investigated. In Figure 10a, the colour gradient helps visualize the height distribution, with the highest peaks marked in red and the deepest valleys in blue. The 3D surface topology reveals a varied surface, with heights ranging from 0 to 308.92 μm, indicating significant surface roughness. The highly irregular fracture surface is likely due to poor adhesion, which can lead to debonding at the PLA–wax interface, resulting in a rough and uneven surface. Poor adhesive qualities between wax and PLA are the primary mechanism for bending fracture. Fracture initiation occurs along the wax and stearic acid network, which coats the Mg-Ca powder. In Figure 10b, the fracture surface shows regions where embedded Mg-Ca powder and pores are concentrated. The porosity is attributed to the pores left by the detached Mg powder during the bending test. These pores and embedded magnesium particles have a significant impact on the fracture behaviour.

### 3.8. Effect of Mg-Ca Volume Content, Particle Size, and Morphology on the Viscosity of Mg-Ca/PLA Composite Filaments

The rheological behaviour of a suspension plays a crucial role in the manufacturing process, directly impacting the properties of the final composite product. Suspensions, as heterogeneous mixtures, consist of solid particles dispersed within a fluid. Factors such as particle volume fraction, size, and shape significantly affect the suspension’s rheological properties. Specifically, a high volume fraction, smaller particle size, and irregular shapes tend to increase the suspension’s viscosity, making it challenging to achieve rapid printing. This increase is attributed to the larger total surface area of the particles, which enhances their interaction with the surrounding fluid [38]. Conversely, poor adhesion between the particles and the fluid can reduce this effect, as non-adhesive particles may act as lubricants [32].

**Figure 11:**
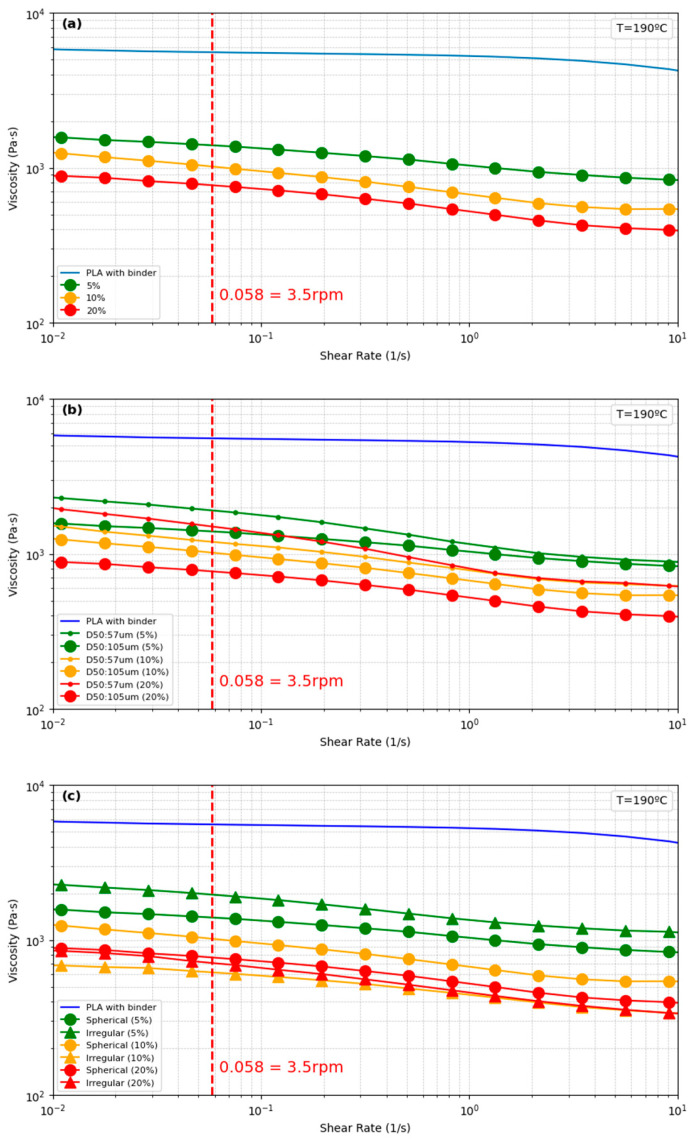
Viscosity as function of shear rate. (**a**) Effect of the Mg-Ca volume contents, (**b**) effect of Mg-Ca particle size, and (**c**) effect of particle morphology.

Viscosity trends in a PLA blend, composed of 90 wt.% PLA, 9 wt.% paraffin wax, and 1 wt.% stearic acid, with different volume fractions of Mg-Ca, are exhibited in Figure 11a. The melted composites comprised a PLA-based blend without Mg-Ca powder and a PLA-based blend with 5%, 10%, and 20% volume fractions of Mg-Ca powder. The composites exhibit shear-thinning behaviour, where viscosity decreases with increasing shear rate [39]. The shear-thinning behaviour enhances the flow of melted composites at elevated shear rates, thereby reducing the requisite pressure in the extrusion process and enhancing the energy efficiency. Additionally, this behavior enables higher 3D printing speeds. The sample of PLA-based blend without Mg-Ca powder exhibits the highest viscosity throughout the entire shear rate spectrum, suggesting that the addition of the Mg-Ca powder reduces the viscosity of the PLA-based blend. At a shear rate of 0.058 1/s, indicated by a red dashed line (Figure 11) representing an optimal rotational speed of 3.5 rpm in filament manufacturing, the viscosities are ranked in descending order as follows: 0 vol.%; 5 vol.%; 10 vol.%; and 20 vol.%. This pattern suggests that the Mg-Ca particles act as a lubricant, indicating that the Mg-Ca powder did not adhere well to the PLA. This pattern suggests that the Mg-Ca particles did not adhere well to the PLA. This could be attributed to the influence of paraffin wax, which is non-polar. It is hypothesised that the paraffin wax coated on Mg-Ca particles reduces the adhesion between Mg-Ca powder particles and PLA, which is relatively polar, though further investigation is required to confirm this. The poor adhesion between the powder and polymer binder can be unfavourable for flexural strength of the composite, but it can improve the processibility in extrusion and printing by reducing the viscosity of the melted composite.

As the volume fraction of Mg-Ca powder increases, PLA flow is expected to be hindered if there is interaction between PLA and powder. Conversely, with minimal interaction between PLA and Mg-Ca particles, the Mg-Ca particles acted as a lubricant within the melted composite, thereby reducing PLA–PLA interaction and enhancing PLA chain mobility. The findings suggest that paraffin wax has an adverse effect on adhesion between Mg-Ca particles and PLA. As stated previously, using non-polar polymers such as polypropylene (PP) or polyethylene (PE) can be implemented to overcome this issue. Specifically, PE exhibits good rheological behavior and miscibility with paraffin wax and stearic acid components. However, PE is not ideal binder because it may cause the formation of Mg-carbonate during the sintering process. PP demonstrated good sintering performance, whereas PP has relatively poor miscibility with paraffin wax compared to PE. A copolymer of pp and pe showed good miscibility and sintering performance in MIM [40,41,42]. Nevertheless, the copolymer of pp and pe at high powder contents does not exhibit sufficient mechanical properties such as elongation for filament winding. To solve this issue, pp and pe copolymer was mixed with elastomer to improve the flexibility of the filament [16]. Alternatively, screw-based 3D printing technique was employed to skip the filament extrusion process [43].

Figure 11b shows the influence of Mg-Ca particle size on PLA composite viscosity. The graph illustrates the effect of particle size on the viscosity of PLA composite materials at a constant temperature of 190 °C. All samples exhibit shear-thinning behaviour. Samples with smaller particles (D50: 57 μm) exhibit higher viscosities compared to those with larger particles (D50: 105 μm) at 5%, 10%, and 20% volume fractions of Mg-Ca powder. This is because smaller particles have a larger surface area, leading to better interaction with the polymer. An increase in particle size results in lower viscosity, indicating a less constrained flow within the PLA matrix. Practical 3D-printing will require higher levels of Mg-powder content (>50%), however the know-how developed in this study with the effects of low levels of Mg-powder (5–20%) will be used as a baseline for future work.

Figure 11c presents the impact of Mg-Ca particle shape on the viscosity in PLA composites, comparing spherical with irregularly shaped particles at various volume fractions (5%, 10%, and 20%). Consistent with previous findings, all composites exhibit shear-thinning behaviour. At a shear rate of 0.058 1/s, composites containing spherical particles show lower viscosities at a 5% volume fraction compared to those with irregular-shaped particles. Conversely, at higher volume fractions (10% and 20%), the composite with irregular-shaped particles show reduced viscosity. The Mg-Ca particles coated with paraffin wax function as a lubricant within the PLA matrix, complicating the viscosity’s dependency on particle sphericity. Furthermore, the difference in roundness between spherical and irregular particles is not large enough. To elucidate the specific mechanisms and see clear differences, further experimental investigation is required.

To summarise, this research emphasises the importance of particle volume content, size, and morphology in determining the mechanical and rheological properties of Mg-Ca/PLA composites. These findings are instrumental in advancing low-temperature additive manufacturing by optimizing filament properties. Furthermore, the optimized filament can enable 3D printing of magnesium implants at low cost, with rapid and patient-specific design capabilities. Future research should focus on enhancing powder-matrix interactions and exploring the potential of alternative blend materials to address issues like porosity and mechanical strength. Further investigations into the rheological behaviour of these composites will also be critical for refining manufacturing processes and expanding their applicability in biomedical applications.

## 4. Conclusions

The characterisation of how Mg alloy powder influences the extrudability of Mg-Ca/PLA composite filaments is essential for material extrusion additive manufacturing. This study systematically investigated the impact of volume content, size, and morphology of Mg-Ca particles on the bending strength and viscosity of PLA composite filaments. The main conclusions can be drawn as follows:

The results demonstrated a pronounced effect of Mg-Ca volume content on the mechanical properties of Mg-Ca/PLA composite filaments. Specifically, an increase in the volume content was observed to correlate with a decrease in the bending strength. This phenomenon is likely due to poor adhesion between the particles and the polymer matrix, and a decrease in volume percentage of the PLA component. Furthermore, the study revealed a trend in viscosity characteristics, where higher Mg-Ca powder volume fractions resulted in lower viscosities of the composites. This behaviour can be attributed to the imperfect particle–matrix interaction. A key factor contributing to this effect is the poor interactions between the paraffin wax and PLA, which disrupts the integrity of the particle–matrix interface and consequently impacts the mechanical stability and flow properties of the composite filaments.

Particle size was found to be a key determinant in the performance of Mg-Ca/PLA composite filaments. Smaller particles (D50: 57 μm) consistently exhibited superior resistance to bending strength. This enhanced mechanical strength is attributed to a more effective stress distribution throughout the PLA matrix. Conversely, larger particles (D50: 105 μm) were linked to a decrease in the composite’s viscosity.

Similarly, the morphology of the powder particles played a critical role, with spherical particles outperforming irregular ones in bending strength. This is likely due to the uniform geometry of spherical particles, which facilitates better stress distribution and potentially stronger particle–matrix bonding. In terms of viscosity, composites with spherical particles exhibited lower viscosities at lower volume fractions, but this trend reversed at higher volume fractions, highlighting the complex interplay between particle shape and composite rheology.

## Figures and Tables

**Table 1:** Parameters for ball milling process to enhance powder sphericity.

Parameter (Unit)	Value	Materials
Duration (h)	4	-
Revolutions per minute (RPM)	650	-
Ball size (mm)	10	Stainless steel
Ball-to-powder ratio	10:1	-

**Table 2:** Design of experiments (DOE) of powder preparation for filament extrusion.

Sample Number	Volume Ratio Mg-Ca in PLA (%)	D50 of Mg-Ca Powder (μm)	Morphology of Mg-Ca Powder
1	5	105	Spherical
2	5	105	Irregular
3	5	57	Spherical
4	5	57	Irregular
5	10	105	Spherical
6	10	105	Irregular
7	10	57	Spherical
8	10	57	Irregular
9	20	105	Spherical
10	20	105	Irregular
11	20	57	Spherical
12	20	57	Irregular

## Data Availability

The data that supports the findings of this study are available from the corresponding author.

## References

[1] Kaushik V., Kumar N., Vignesh M. (2022). Magnesium role in additive manufacturing of biomedical implants—Challenges and opportunities. Addit. Manuf..

[2] Tappa K., Jammalamadaka U. (2018). Novel Biomaterials Used in Medical 3D Printing Techniques. J. Funct. Biomater..

[3] Fernandes H.R., Gaddam A., Rebelo A., Brazete D., Stan G.E., Ferreira J.M. (2018). Bioactive Glasses and Glass-Ceramics for Healthcare Applications in Bone Regeneration and Tissue Engineering. Materials.

[4] Li M., Benn F., Derra T., Kröger N., Zinser M., Smeets R., Molina-Aldareguia J.M., Kopp A., LLorca J. (2021). Microstructure, mechanical properties, corrosion resistance and cytocompatibility of WE43 Mg alloy scaffolds fabricated by laser powder bed fusion for biomedical applications. Mater. Sci. Eng. C Mater. Biol. Appl..

[5] Li Z., Gu X., Lou S., Zheng Y. (2008). The development of binary Mg-Ca alloys for use as biodegradable materials within bone. Biomaterials.

[6] Wang Y., Huang H., Jia G., Zeng H., Yuan G. (2021). Fatigue and dynamic biodegradation behavior of additively manufactured Mg scaffolds. Acta Biomater..

[7] Li Y., Zhou J., Pavanram P., Leeflang M.A., Fockaert L.I., Pouran B., Tümer N., Schröder K.U., Mol J.M.C., Weinans H. (2018). Additively manufactured biodegradable porous magnesium. Acta Biomater..

[8] Nasr Azadani M., Zahedi A., Bowoto O.K., Oladapo B.I. (2022). A review of current challenges and prospects of magnesium and its alloy for bone implant applications. Prog. Biomater..

[9] Liu C., Lu S., Fu Y., Zhang H. (2015). Flammability and the oxidation kinetics of the magnesium alloys AZ31, WE43, and ZE10. Corros. Sci..

[10] Hu D., Wang Y., Zhang D., Hao L., Jiang J., Li Z., Chen Y. (2015). Experimental Investigation on Selective Laser Melting of Bulk Net-Shape Pure Magnesium. Mater. Manuf. Process..

[11] Chung Ng C., Savalani M., Chung Man H. (2011). Fabrication of magnesium using selective laser melting technique. Rapid Prototyp. J..

[12] Gonzalez-Gutierez J., Godec D., Guráň R., Spoerk M., Kukla C., Holzer C. (2018). 3D printing conditions determination for feedstock used in fused filament fabrication (FFF) of 17-4PH stainless steel parts. Metalurgija.

[13] Gonzalez-Gutierrez J., Cano S., Schuschnigg S., Kukla C., Sapkota J., Holzer C. (2018). Additive Manufacturing of Metallic and Ceramic Components by the Material Extrusion of Highly-Filled Polymers: A Review and Future Perspectives. Materials.

[14] Agarwala M.K., Weeren R.V., Vaidyanathan R., Bandyopadhyay A., Carrasquillo G., Jamalabad V., Langrana N., Safari A., Garofalini S.H., Danforth S.C. Structural Ceramics by Fused Deposition of Ceramics. Proceedings of the International Solid Freeform Fabrication Symposium.

[15] Antoniac I., Popescu D., Zapciu A., Antoniac A., Miculescu F., Moldovan H. (2019). Magnesium Filled Polylactic Acid (PLA) Material for Filament Based 3D Printing. Materials.

[16] Wolff M., Mesterknecht T., Bals A., Ebel T., Willumeit-Römer R. (2019). FFF of Mg-Alloys for Biomedical Application. Magnesium Technology.

[17] Wagner M.A., Hadian A., Sebastian T., Clemens F., Schweizer T., Rodriguez-Arbaizar M., Carreno-Morelli E., Spolenak R. (2022). Fused filament fabrication of stainless steel structures-from binder development to sintered properties. Addit. Manuf..

[18] Kukla C., Gonzalez-Gutierrez J., Duretek I., Schuschnigg S., Holzer C. Effect of particle size on the properties of highly-filled polymers for fused filament fabrication. Proceedings of the 32nd International Conference of the Polymer Processing Society.

[19] Wu G., Langrana N.A., Rangarajan S., McCuiston R., Sadanji R., Danforth S., Safari A. Fabrication of Metal Components using FDMet: Fused Deposition of Metals. Proceedings of the Solid Freeform Fabrication Symposium.

[20] Kukla C., Gonzalez-Gutierrez J., Cano S.C., Hampel S., Burkhardt C., Moritz T., Holzer C. Fused filament fabrication (FFF) of PIM feedstocks. Proceedings of the VI Congreso Nacional de Pulvimetalurgia y I Congreso Iberoamericano de Pulvimetalurgia.

[21] Maurel A., Kim H., Russo R., Grugeon S., Armand M., Panier S., Dupont L. (2021). Ag-Coated Cu/Polylactic Acid Composite Filament for Lithium and Sodium-Ion Battery Current Collector Three-Dimensional Printing via Thermoplastic Material Extrusion. Front. Energy Res..

[22] Celikin M., Azadi A., Kim H., Vaughan T., O’Cearbhaill E. (2023). Development of Magnesium-Strontium/Calcium (Mg-Sr/Ca)-Based Alloys with Improved Sinterability for Next-Generation Biomedical Implants. Proceedings of the TMS Annual Meeting & Exhibition, San Diego, CA, USA, 19–23 March 2023.

[23] (2017). Standard Test Methods for Flexural Properties of Unreinforced and Reinforced Plastics and Electrical Insulating Materials.

[24] (2023). Standard Test Method for Plastics: Dynamic Mechanical Properties Melt Rheology.

[25] Silva A.N., Cipriano T., Silva H.M., Rocha M.C.C.G., Maria F.A., Monteiro G. (2014). Thermal, rheological and morphological properties of poly (lactic acid) (PLA) and talc composites. Polímeros Ciência Tecnol..

[26] Kervran M., Vagner C., Cochez M., Ponçot M., Saeb M.R., Vahabi H. (2022). Thermal degradation of polylactic acid (PLA)/polyhydroxybutyrate (PHB) blends: A systematic review. Polym. Degrad. Stab..

[27] Rasal R.M., Janorkar A.V., Hirt D.E. (2010). Poly(lactic acid) modifications. Prog. Polym. Sci..

[28] Naser A.Z., Deiab I., Darras B.M. (2021). Poly(lactic acid) (PLA) and polyhydroxyalkanoates (PHAs), green alternatives to petroleum-based plastics: A review. RSC Adv..

[29] Ma J., Qin M., Zhang L., Tian L., Li R., Chen P., Qu X. (2014). Effect of ball milling on the rheology and particle characteristics of Fe–50%Ni powder injection molding feedstock. J. Alloys Compd..

[30] Jardiel T., Levenfeld B., Jimenez R., Varez A. (2009). Fabrication of 8-YSZ thin-wall tubes by powder extrusion moulding for SOFC electrolytes. Ceram. Int..

[31] Fu S.Y., Feng X.Q., Lauke B., Mai Y.W. (2008). Effects of particle size, particle/matrix interface adhesion and particle loading on mechanical properties of particulate–polymer composites. Compos. Part. B Eng..

[32] Ghasemi-Mobarakeh L., Cano S., Momeni V., Liu D., Duretek I., Riess G., Kukla C., Holzer C. (2022). Effect of Increased Powder-Binder Adhesion by Backbone Grafting on the Properties of Feedstocks for Ceramic Injection Molding. Polymers.

[33] Wolff M., Schaper J.G., Dahms M., Ebel T., Kainer K.U., Klassen T. (2014). Magnesium powder injection moulding for biomedical application. Powder Metall..

[34] Ebel T. (2018). Titanium MIM for manufacturing of medical implants and devices. Titanium in Medical and Dental Applications.

[35] Suwanpreecha C., Manonukul A. (2022). A Review on Material Extrusion Additive Manufacturing of Metal and How It Compares with Metal Injection Moulding. Metals.

[36] Zhang Q., Tian M., Wu Y., Lin G., Zhang L. (2004). Effect of particle size on the properties of Mg(OH)_2_-filled rubber composites. J. Appl. Polym. Sci..

[37] Tzetzis D., Hogg P.J. (2007). The influence of surface morphology on the interfacial adhesion and fracture behavior of vacuum infused carbon fiber reinforced polymeric repairs. Polym. Compos..

[38] Hao X., Kaschta J., Schubert D.W. (2016). Viscous and elastic properties of polylactide melts filled with silica particles: Effect of particle size and concentration. Compos. Part B Eng..

[39] Hasib A.G., Niauzorau S., Xu W., Niverty S., Kublik N., Williams J., Chawla N., Song K., Azeredo B. (2021). Rheology scaling of spherical metal powders dispersed in thermoplastics and its correlation to the extrudability of filaments for 3D printing. Addit. Manuf..

[40] Wolff M., Schaper J.G., Suckert M.R., Dahms M., Feyerabend F., Ebel T., Willumeit-Römer R., Klassen T. (2016). Metal Injection Molding (MIM) of Magnesium and Its Alloys. Metals.

[41] Wolff M., Schaper J.G., Suckert M.R., Dahms M., Ebel T., Willumeit-Römer R., Klassen T. (2016). Magnesium Powder Injection Molding (MIM) of Orthopedic Implants for Biomedical Applications. Jom.

[42] Schaper J.G., Wolff M., Wiese B., Ebel T., Willumeit-Römer R. (2019). Powder metal injection moulding and heat treatment of AZ81 Mg alloy. J. Mater. Process. Technol..

[43] Wolff M., Marvi-Mashhadi M., Nidadavolu E., Lüneburg H., Ebel T., Willumeit-Römer R. (2023). Comparison between compression tested and simulated Mg-6.3Gd bone scaffolds produced by binder based additive manufacturing technique. J. Magnes. Alloys.

